# Inflammatory–lipid residual risk phenotypes delineate the HFpEF probability spectrum independent of cardiometabolic mediation

**DOI:** 10.1186/s12872-026-05836-3

**Published:** 2026-04-29

**Authors:** Chunmei Chen, Siqi Zhang, Yuetong Liu, Lu Zhao, Yuxin Wang, Yumeng Li, Qingqiao Song

**Affiliations:** 1 Department of General Internal Medicine, Guang’anmen Hospital, China Academy of Chinese Medical Sciences, Beijing, China; 2 College of Traditional Chinese Medicine, China Academy of Chinese Medical Sciences, Beijing, China; 3 Beijing University of Chinese Medicine, Beijing, China

**Keywords:** HFpEF, HFA-PEFF, Residual inflammatory risk, Residual cholesterol risk, Inflammatory-lipid residual risk phenotype

## Abstract

**Background:**

Heart failure with preserved ejection fraction (HFpEF) is increasingly recognized as a systemic syndrome characterized by chronic low-grade inflammation, metabolic dysregulation, and microvascular dysfunction. Although residual inflammatory risk and residual cholesterol risk have each been associated with adverse cardiovascular outcomes, their joint relationship with HFpEF probability spectrum, and the extent to which this relationship is mediated by conventional cardiometabolic pathways, remain unclear.

**Methods:**

In this cross-sectional study, participants were classified into four inflammatory-lipid residual risk phenotypes according to low-density lipoprotein cholesterol (LDL-C) and high-sensitivity C-reactive protein (hs-CRP): no residual risk, residual inflammatory risk (RIR), residual cholesterol risk (RCR), and combined residual inflammatory and cholesterol risk (RCIR). HFpEF probability was assessed using the Heart Failure Association Pre-test assessment, Echocardiography and natriuretic peptide, Functional testing, Final etiological work-up (HFA-PEFF) score and categorized as low (≤ 1), intermediate (2–4), or high (≥ 5). Associations between residual risk phenotypes and the HFpEF probability spectrum were examined using ordinal logistic regression and marginal predicted probabilities analyses. Exploratory mediation analyses were performed to evaluate the contribution of conventional cardiometabolic components.

**Results:**

Residual risk phenotypes were distributed heterogeneously across HFA-PEFF strata. The proportion of RCIR increased progressively with higher HFpEF probability, accounting for 26.4% in the low stratum, 34.9% in the intermediate stratum, and 43.5% in the high stratum, whereas the proportion of RCR decreased from 68.4% to 60.8% and 46.7%, respectively (*P* < 0.001). In the fully adjusted ordinal logistic model, RCIR (OR 0.59, 95% CI 0.41–0.84), RIR (OR 0.53, 95% CI 0.32–0.90), and particularly RCR (OR 0.38, 95% CI 0.27–0.54) were associated with lower odds of remaining in lower HFA-PEFF categories, consistent with a shift toward higher HFpEF probability strata. Marginal predicted probability analyses across Models 1–3 showed a consistent redistribution of HFA-PEFF probability across phenotypes, and the overall ordering pattern remained stable after full adjustment. Exploratory mediation analyses showed no meaningful mediation by BMI or systolic blood pressure, while glucose demonstrated only a modest borderline signal. Overall, the association between RCIR and higher HFpEF probability appeared not to be materially mediated by the selected conventional cardiometabolic variables.

**Conclusions:**

Inflammatory-lipid residual risk phenotypes were differentially associated with the HFpEF probability spectrum. RCIR showed progressive enrichment with increasing HFpEF probability, whereas RCR exhibited an inverse distributional pattern but remained strongly associated with higher-probability strata in multivariable models. These associations were largely independent of conventional cardiometabolic mediators, supporting a potential role for inflammatory-lipid residual risk phenotyping in refining HFpEF risk stratification.

**Supplementary Information:**

The online version contains supplementary material available at https://doi.org/10.1186/s12872-026-05836-3.

## Introduction

HFpEF has become the dominant form of heart failure worldwide and remains a major unmet clinical challenge [1, 2]. It is characterized by substantial clinical and biological heterogeneity, a high burden of comorbidities, and limited therapeutic options. Increasing evidence suggests that HFpEF should be understood not simply as a cardiac disorder, but as a systemic syndrome involving chronic low-grade inflammation [3], metabolic dysfunction [4], endothelial impairment [5], and microvascular remodeling [6].

Inflammation has been increasingly recognized as a central contributor to HFpEF pathophysiology. Elevated inflammatory biomarkers, including hs-CRP, IL-6, and TNF-α, have been associated with incident heart failure, adverse myocardial remodeling, and poor prognosis in HFpEF [7, 8]. In parallel, dyslipidemia remains a cornerstone of cardiovascular risk assessment and management [9]. Even when LDL-C is reduced to guideline-recommended levels, a substantial proportion of individuals continue to experience adverse cardiovascular or cardiometabolic events, highlighting the concept of residual cholesterol risk. Emerging evidence further suggests that lipid-related residual risk often coexists with low-grade systemic inflammation, underscoring the need for integrated inflammatory-lipid phenotyping rather than reliance on isolated lipid measures alone [10].

Residual risk is increasingly viewed as a multidimensional construct that includes both inflammatory and lipid-related components [11]. RIR reflects persistent inflammatory activity despite lower LDL-C, whereas RCR reflects continued atherogenic burden despite lower inflammatory activity[12]. Although both constructs have been extensively studied in atherosclerotic cardiovascular disease [13–15], their relevance to HFpEF, especially when considered jointly, remains insufficiently characterized.

Most previous studies have examined inflammation and lipid abnormalities separately, implicitly treating these pathways as parallel or additive contributors to disease [16–20]. Whether their coexistence identifies a distinct phenotype linked to higher HFpEF probability has not been systematically explored. In addition, it remains unclear whether any association between inflammatory-lipid residual risk and HFpEF is primarily mediated through conventional cardiometabolic pathways, such as obesity, hypertension, and dysglycemia, or whether it reflects predominantly direct pathophysiological effects.

The Heart Failure Association–Pre-test assessment, Echocardiography and natriuretic peptide, Functional testing, HFA-PEFF score provides a validated framework for estimating HFpEF probability [21]. Rather than imposing a binary diagnosis, it stratifies patients into ordinal probability categories, thereby offering a useful tool for phenotypic investigation across different levels of HFpEF likelihood.

**Fig. 1:**
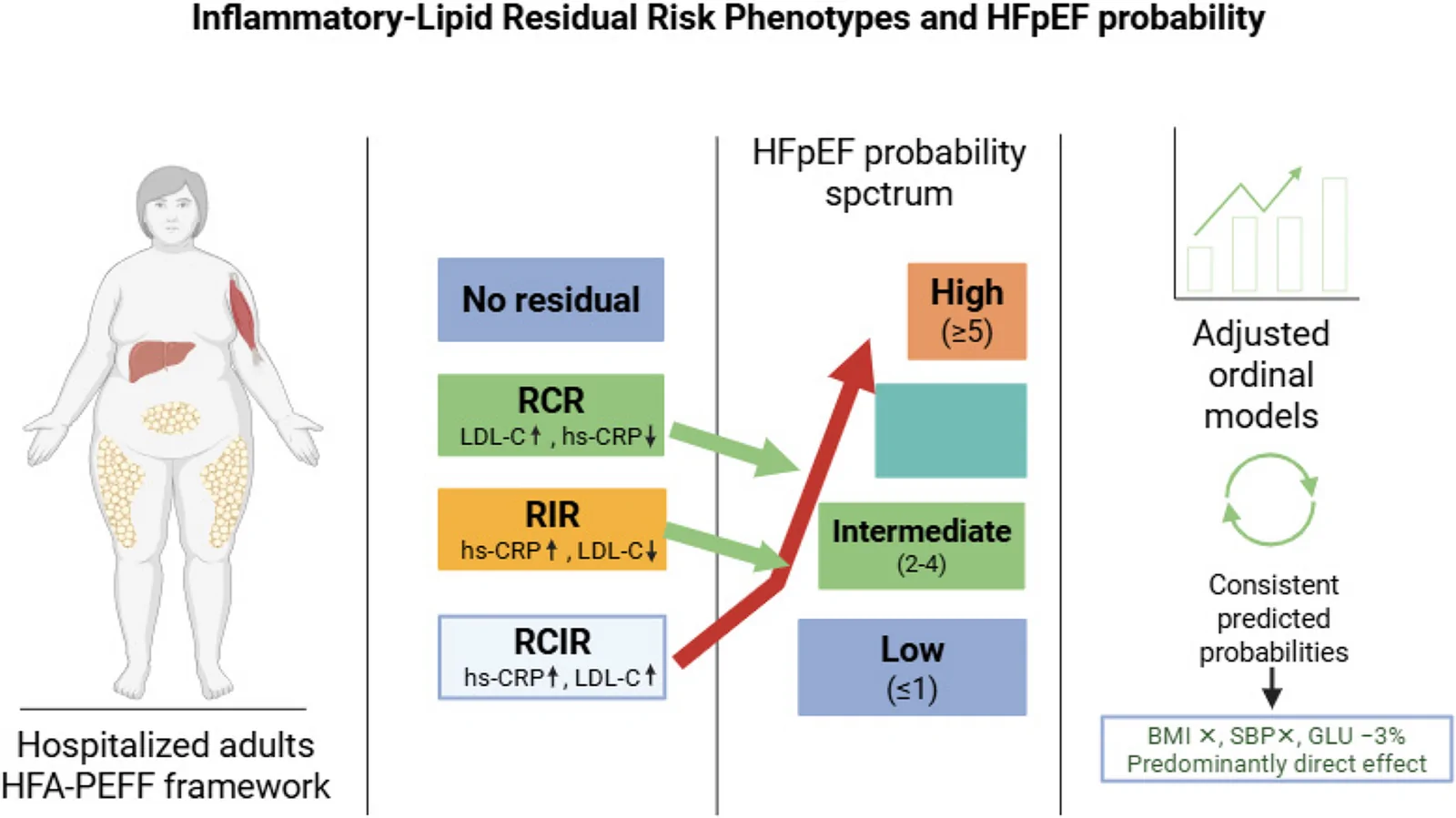
Graphical abstract. Inflammatory–lipid residual risk phenotypes defined by hs-CRP and LDL-C were differentially distributed across the HFA-PEFF–derived HFpEF probability spectrum. RCIR and RIR were associated with higher HFpEF probability, whereas RCR predominated in lower probability strata. Associations were assessed using adjusted ordinal regression and marginal predicted probability analyses

In this study, we investigated the association between inflammatory-lipid residual risk phenotypes and the HFpEF probability categories. We further performed exploratory mediation analyses to assess whether conventional cardiometabolic components mediate these associations, with the aim of clarifying potential links among inflammation, lipid dysregulation, and HFpEF (Fig. 1). 

## Methods

### Study design and population

This was a single-center, cross-sectional study conducted in the Department of General Medicine, Guang’anmen Hospital, China Academy of Chinese Medical Sciences, between September 2021 and April 2024. Adult inpatients who underwent assessments of lipid parameters, inflammatory biomarkers, and echocardiographic examination during the same hospitalization were screened for eligibility. Participants were required to have sufficient clinical, laboratory, and echocardiographic data to support HFA-PEFF scoring and inflammatory-lipid residual risk phenotyping. A flow diagram of participant selection is provided in Figure S1.

### Eligibility criteria

Participants were eligible if they met all of the following criteria: (1) age ≥ 18 years; (2) availability of lipid profile and inflammatory biomarker measurements; and (3) availability of the clinical and echocardiographic data required for HFA-PEFF assessment. Exclusion criteria were: (1) severe cardiovascular or cerebrovascular conditions, such as cardiogenic shock or acute intracerebral hemorrhage; (2) acute infection; (3) severe hepatic dysfunction, defined as alanine aminotransferase or aspartate aminotransferase levels ≥ 3 times the upper limit of normal, malignant tumors, or autoimmune diseases; (4) pregnancy or planned pregnancy; and (5) absence of core data required for exposure definition or outcome assessment.

### Assessment of HFpEF probability

**Fig. 2:**
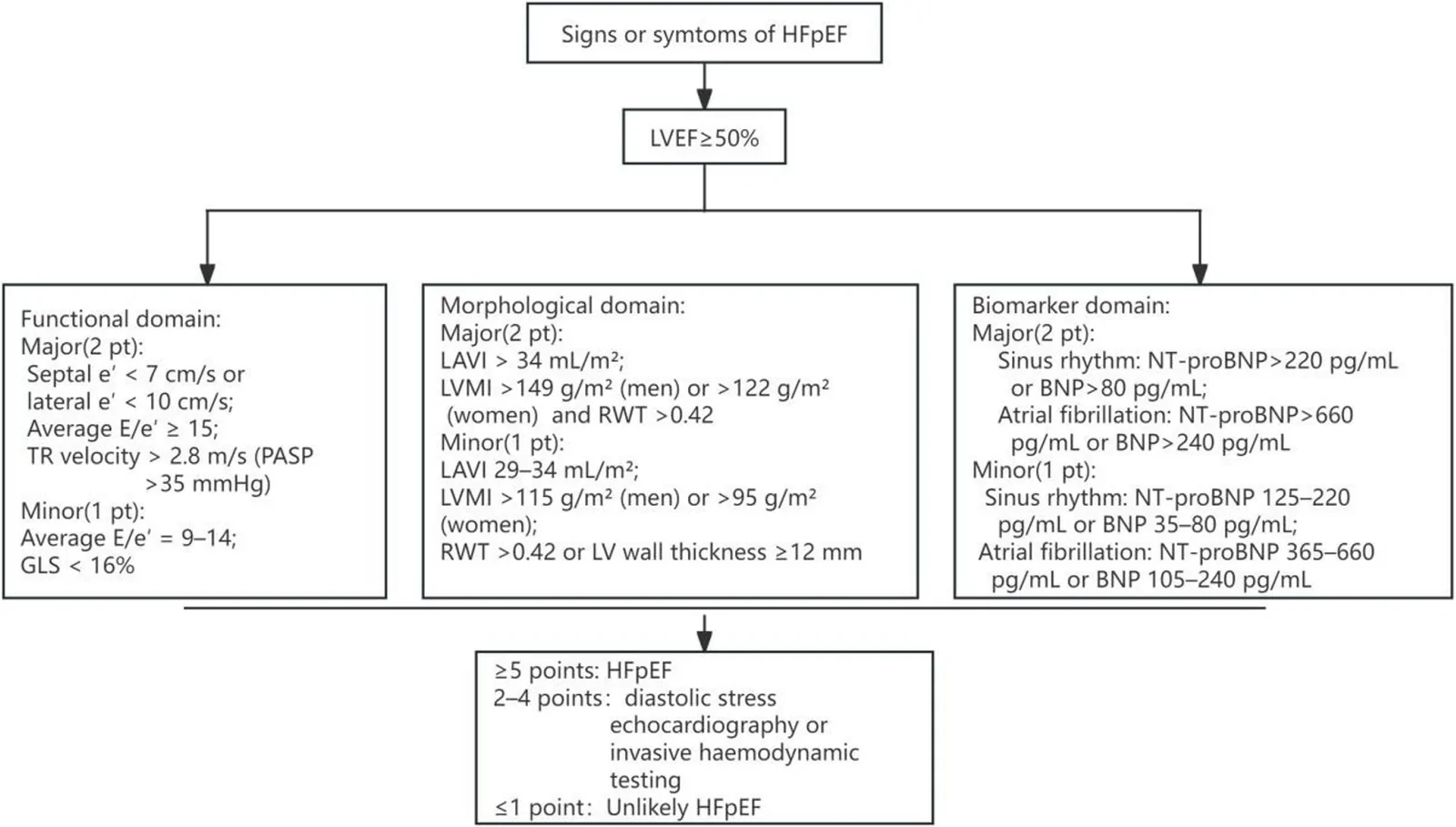
HFA-PEFF diagnostic algorithm for assessment of HFpEF probability. The HFA-PEFF score integrates three domains including functional, morphological, and biomarker to quantify the likelihood of HFpEF in patients with signs or symptoms of heart failure and left ventricular ejection fraction ≥50%. Major and minor criteria within each domain are assigned 2 and 1 point(s), respectively. The total score ranges from 0 to 6 points and classifies individuals into three ordered categories: low probability (≤1 point), intermediate probability (2-4 points), and high probability (≥5 points) of HFpEF

HFpEF probability was assessed using the HFA-PEFF score. In brief, participants with signs and/or symptoms suggestive of heart failure and preserved left ventricular ejection fraction (LVEF ≥ 50%) were evaluated across functional, morphological, and biomarker domains according to the published HFA-PEFF recommendations, with NT-proBNP interpreted according to rhythm status. Echocardiographic parameters and laboratory measurements used for HFA-PEFF scoring were obtained during the same hospitalization. Participants were categorized as low (≤ 1), intermediate (2–4), or high (≥ 5) probability of HFpEF according to the recommended score thresholds. This ordered classification served as the primary outcome (Fig. 2). 

### Definition of inflammatory–lipid residual risk phenotypes

Inflammatory-lipid residual risk phenotypes were defined operationally using LDL-C and hs-CRP thresholds. LDL-C < 1.8 mmol/L was considered lower lipid-risk status, whereas hs-CRP ≥ 2 mg/L was considered elevated inflammatory activity. hs-CRP values were defined using the first blood sample obtained after hospital admission. Participants were classified into four mutually exclusive phenotypes: no residual risk (LDL-C < 1.8 mmol/L and hs-CRP < 2 mg/L); RIR (LDL-C < 1.8 mmol/L and hs-CRP ≥ 2 mg/L); RCR (LDL-C ≥ 1.8 mmol/L and hs-CRP < 2 mg/L); and combined RCIR (LDL-C ≥ 1.8 mmol/L and hs-CRP ≥ 2 mg/L).

### Covariates

Covariates were selected a priori based on clinical relevance and their potential relationships with both residual risk phenotypes and HFpEF probability. Demographic covariates included age and gender. Cardiometabolic covariates included BMI, SBP, GLU, and serum creatinine (Scr). These variables were incorporated into multivariable models to evaluate whether the association between inflammatory-lipid residual risk phenotypes and HFpEF probability categories was independent of conventional cardiometabolic burden.

### Missing data and imputation

Several candidate variables had varying degrees of missingness. To reduce potential bias and account for uncertainty related to missing data, multiple imputation by chained equations (MICE) was performed. The imputation model included all candidate variables used in the analyses. Twenty imputed datasets were generated with 10 iterations per dataset, and the prespecified analyses were conducted separately within each dataset. Regression coefficients, standard errors, and 95% confidence intervals were pooled using Rubin’s rules. Model discrimination was summarized across imputations as the mean and range. For graphical presentation, the first imputed dataset was used as the representative dataset. Missing values in variables contributing to HFA-PEFF scoring were handled within the same multiple-imputation framework.

### Statistical analysis

Continuous variables are presented as mean ± standard deviation or median (interquartile range), as appropriate, and categorical variables as counts (percentages). Group differences were compared using one-way analysis of variance or Kruskal-Wallis tests for continuous variables, and χ² tests for categorical variables. Associations between inflammatory-lipid residual risk phenotypes and HFpEF probability categories were examined using proportional-odds ordinal logistic regression. Model 1 included residual risk phenotype only; Model 2 additionally adjusted for age and gender; Model 3 further adjusted for BMI, SBP, GLU, and Scr. Results were reported as ORs with 95% CIs. ORs < 1 indicate lower odds of remaining in lower HFA-PEFF categories and thus a shift toward higher HFpEF probability strata. Multicollinearity among covariates was assessed using variance inflation factors (VIFs), and no substantial collinearity was identified (all VIFs < 10).

Marginal predicted probabilities were estimated from the ordinal models. The proportional-odds assumption was assessed using score tests and graphical diagnostics. Missing data were handled using multiple imputation by chained equations with pooling by Rubin’s rules. Sensitivity analyses included binary logistic regression models with alternative HFA-PEFF thresholds, multinomial logistic regression, and analyses excluding participants with markedly elevated hs-CRP. Exploratory mediation analyses were conducted for BMI, SBP, GLU and a composite cardiometabolic burden score constructed using nonparametric bootstrapping with 1,000 simulations. All analyses were performed using R software, and a two-sided *P* < 0.05 was considered statistically significant.

## Results

### Clinical characteristics across the HFpEF probability categories

A total of 2,187 participants were included in the final analytical cohort. Participants of low, intermediate, and high probability groups were 348, 637, and 1202 respectively (Table 1). Across increasing HFpEF probability categories, participants were older and exhibited progressively higher systolic blood pressure, fasting glucose, and hs-CRP levels (all P < 0.001). In contrast, LDL-C showed a decreasing trend across categories. BMI and gender distribution did not differ significantly among groups.

**Table 1:** Baseline characteristics across HFA-PEFF probability strata

Variables	level	Low ( < = 1)	Intermediate (2–4)	High ( > = 5)	*P*
n		348	637	1202	
Age		54.27 ± 12.18	64.42 ± 11.74	72.04 ± 11.28	< 0.001
BMI		25.10 ± 4.08	25.19 ± 4.23	25.20 ± 4.11	0.917
SBP		130.13 ± 17.60	135.86 ± 19.48	137.48 ± 19.83	< 0.001
GLU		6.75 ± 3.19	7.28 ± 3.18	7.54 ± 3.24	< 0.001
Scr		63.5 (23)	65.0 (25.0)	70.0 (31.0)	0.253
LDL-C		3.14 ± 0.86	3.05 ± 0.87	2.87 ± 0.87	< 0.001
hs-CRP		1.07 (1.97)	1.45 (2.75)	1.91 (5.52)	< 0.001
Gender	Female	176 (50.6)	343 (53.8)	669 (55.7)	0.236
	Male	172 (49.4)	294 (46.2)	533 (44.3)	
Residual risk phenotype	No residual	8 (2.3)	12 (1.9)	57 (4.7)	< 0.001
	RIR	10 (2.9)	16 (2.5)	61 (5.1)	
	RCR	238 (68.4)	387 (60.8)	561 (46.7)	
	RCIR	92 (26.4)	222 (34.9)	523 (43.5)	

Data are presented as mean ± SD for normally distributed continuous variables, median (IQR) for skewed continuous variables, and n (%) for categorical variables. P values were derived from one-way ANOVA or Kruskal–Wallis tests for continuous variables and χ² tests for categorical variables.

*Abbreviations*: *BMI *body mass index, *Cr *creatinine, *GLU *fasting glucose, *hs-CRP* high-sensitivity C-reactive protein, *LDL-C* low-density lipoprotein cholesterol, *SBP *systolic blood pressure

Overall, higher HFA-PEFF probability was accompanied by a less favorable cardiometabolic and inflammatory profile, characterized by older age, higher blood pressure, poorer glycemic status, and greater systemic inflammatory burden, whereas LDL-C levels tended to be lower in the higher-probability strata. 

### Distribution of residual risk phenotypes across HFA-PEFF categories

**Fig. 3:**
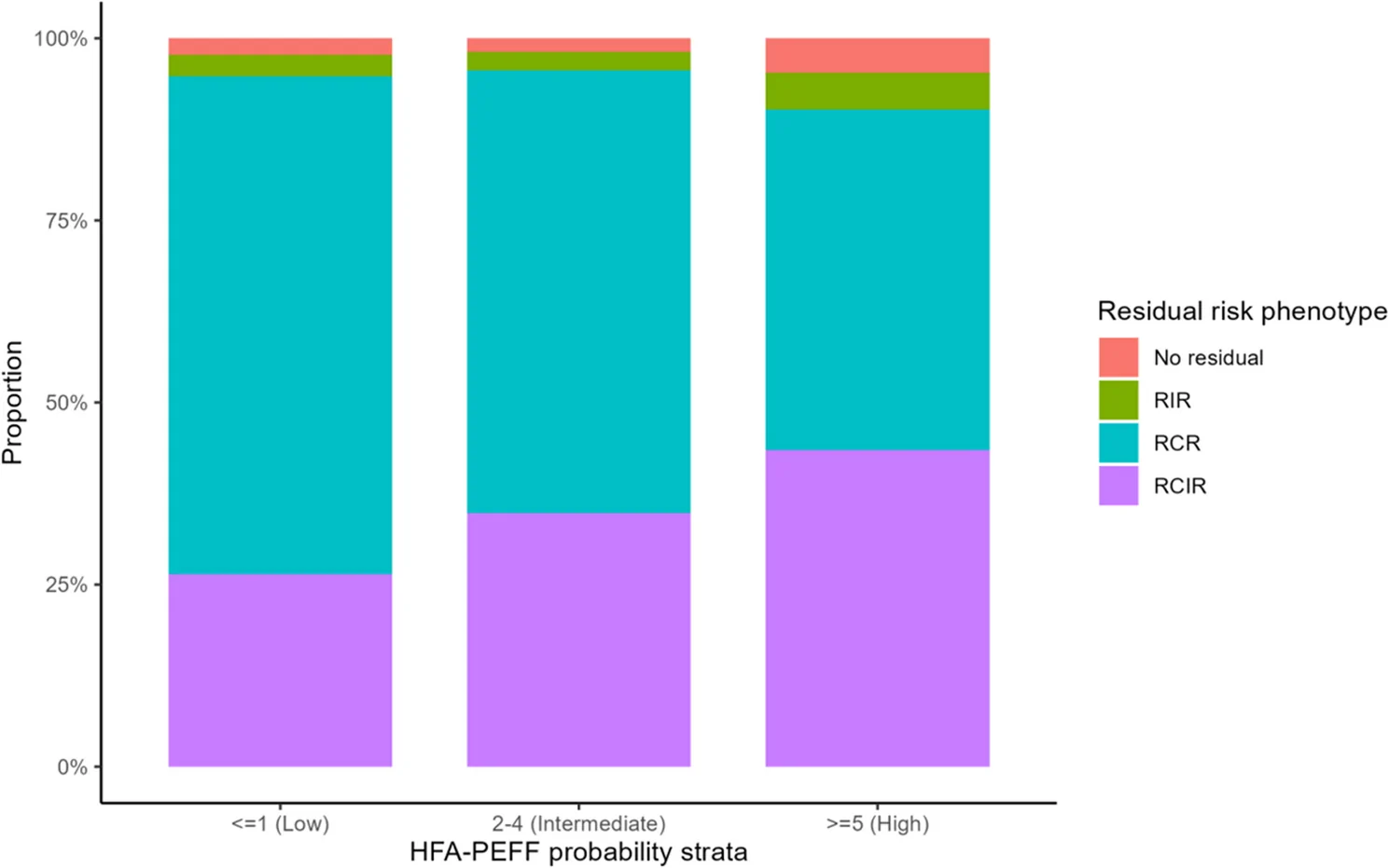
Distribution of inflammatory-lipid residual risk phenotypes across HFA-PEFF probability strata. Stacked bar plot showing the proportional composition of residual risk phenotypes within each HFA-PEFF probability stratum (low ≤1, intermediate 2–4, high ≥5). Residual risk phenotypes include no residual risk, RIR, RCR, and RCIR as defined by LDL-C and hs-CRP thresholds. The figure illustrates systematic redistribution of residual risk phenotypes across increasing HFpEF probability strata

The distribution of inflammatory-lipid residual risk phenotypes differed significantly across HFA-PEFF probability categories (Fig. 3). RCIR showed a progressive increase from 26.4% in the low-probability group to 34.9% in the intermediate-probability group and 43.5% in the high-probability group, whereas RCR decreased from 68.4% to 60.8% and 46.7%, respectively. RIR remained relatively infrequent in the low and intermediate probability groups, accounting for 2.9% and 2.5%, respectively, but increased to 5.1% in the high-probability group. The no-residual-risk phenotype accounted for only a small proportion across all categories (2.3%, 1.9%, and 4.7%). 

### Ordinal association between residual risk phenotypes and HFpEF probability

Associations between inflammatory-lipid residual risk phenotypes and ordered HFA-PEFF probability categories were examined using proportional-odds ordinal logistic regression. Using the no-residual-risk phenotype as the reference, ORs < 1 indicate lower odds of remaining in lower HFA-PEFF categories and therefore correspond to a shift toward higher HFpEF probability categories. In model 1 and 2, RCR showed the most consistent association with higher HFpEF probability, whereas RCIR and RIR were weaker or borderline. In Model 3, both RCIR and RIR became statistically significant, whereas RCR remained the strongest associated phenotype (Table 2). 

**Table 2:** Ordinal logistic regression models for the association between residual risk phenotypes and the HFpEF probability spectrum

model	comparison	OR	CI_low	CI_high	P_value
Model 1	RIR vs. No residual	0.83	0.42	1.63	0.58
Model 1	RCR vs. No residual	0.33	0.20	0.55	< 0.001
Model 1	RCIR vs. No residual	0.62	0.37	1.04	0.07
Model 2	RIR vs. No residual	0.51	0.25	1.06	0.07
Model 2	RCR vs. No residual	0.40	0.23	0.69	< 0.001
Model 2	RCIR vs. No residual	0.62	0.36	1.09	0.09
Model 3	RIR vs. No residual	0.53	0.32	0.90	0.02
Model 3	RCR vs. No residual	0.38	0.27	0.54	< 0.001
Model 3	RCIR vs. No residual	0.59	0.41	0.84	< 0.01

Odds ratios (ORs) and 95% confidence intervals (CIs) were estimated using proportional odds ordinal logistic regression. Model 1 included residual risk phenotype only, Model 2 adjusted for age and sex, Model 3 further adjusted for BMI, SBP, GLU, and Cr. The proportional odds assumption was assessed and not materially violated. HFpEF probability categories were defined according to the HFA-PEFF score

**Fig. 4:**
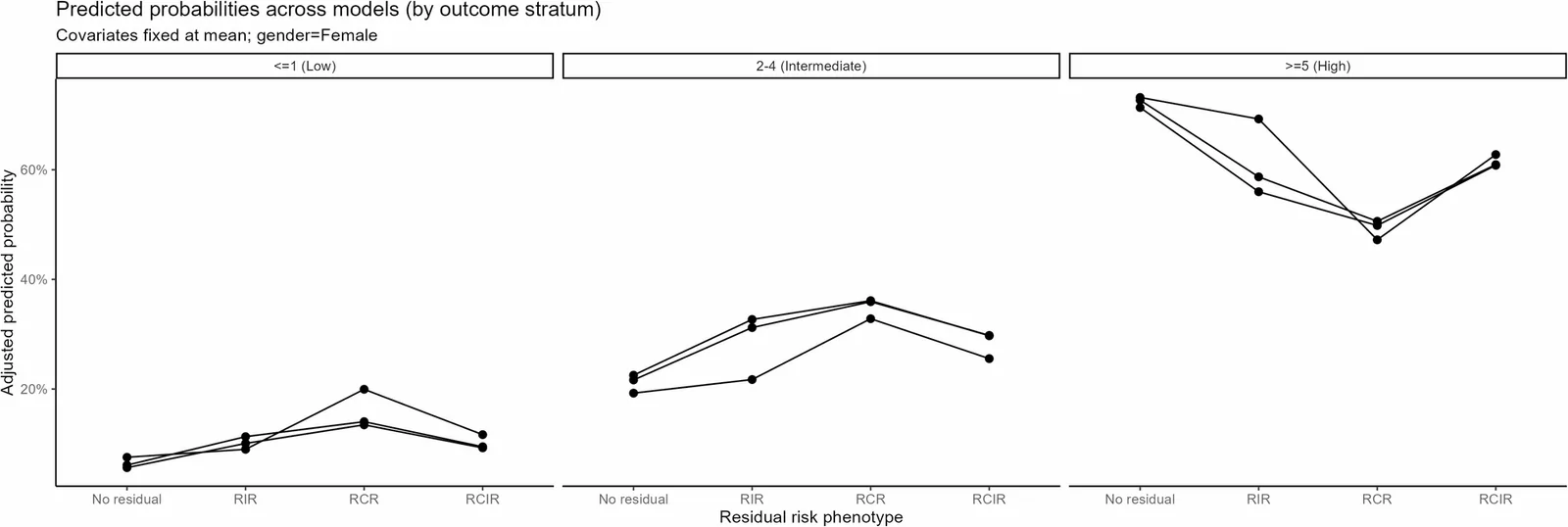
Marginal predicted probabilities of HFA-PEFF categories across residual risk phenotypes. Marginal predicted probabilities of each HFA-PEFF probability stratum (low ≤1, intermediate 2–4, high ≥5) were estimated from ordinal logistic regression models and plotted by residual risk phenotype (no residual, RIR, RCR, RCIR). Model 1 included phenotype only; Model 2 additionally adjusted for age and sex; Model 3 additionally adjusted for BMI, SBP, GLU, and Cr. Predicted probabilities were computed with covariates fixed at their sample means; for visualization, sex was set to female (as labeled in the figure). Lines depict probability redistribution across phenotypes and attenuation after cardiometabolic adjustment

Marginal predicted probabilities derived from Models 1-3 are presented in Fig. 4. The overall ordering pattern across phenotypes was preserved after sequential adjustment, supporting a consistent redistribution toward higher HFpEF probability categories. 

Sensitivity analyses supported the robustness of the primary findings. Excluding participants with hs-CRP > 10 mg/L yielded results that were materially consistent with the main ordinal models (Table S1). Additional binary logistic regression analyses using alternative HFA-PEFF thresholds (high vs. low; intermediate-to-high vs. low) showed similar associations between residual risk phenotypes and HFpEF probability (Table S2). Multinomial logistic regression analyses, performed without the proportional-odds assumption, also showed a comparable overall pattern across HFA-PEFF categories (Table S3).

### Impact of cardiometabolic adjustment and exploratory mediation analyses

**Fig. 5:**
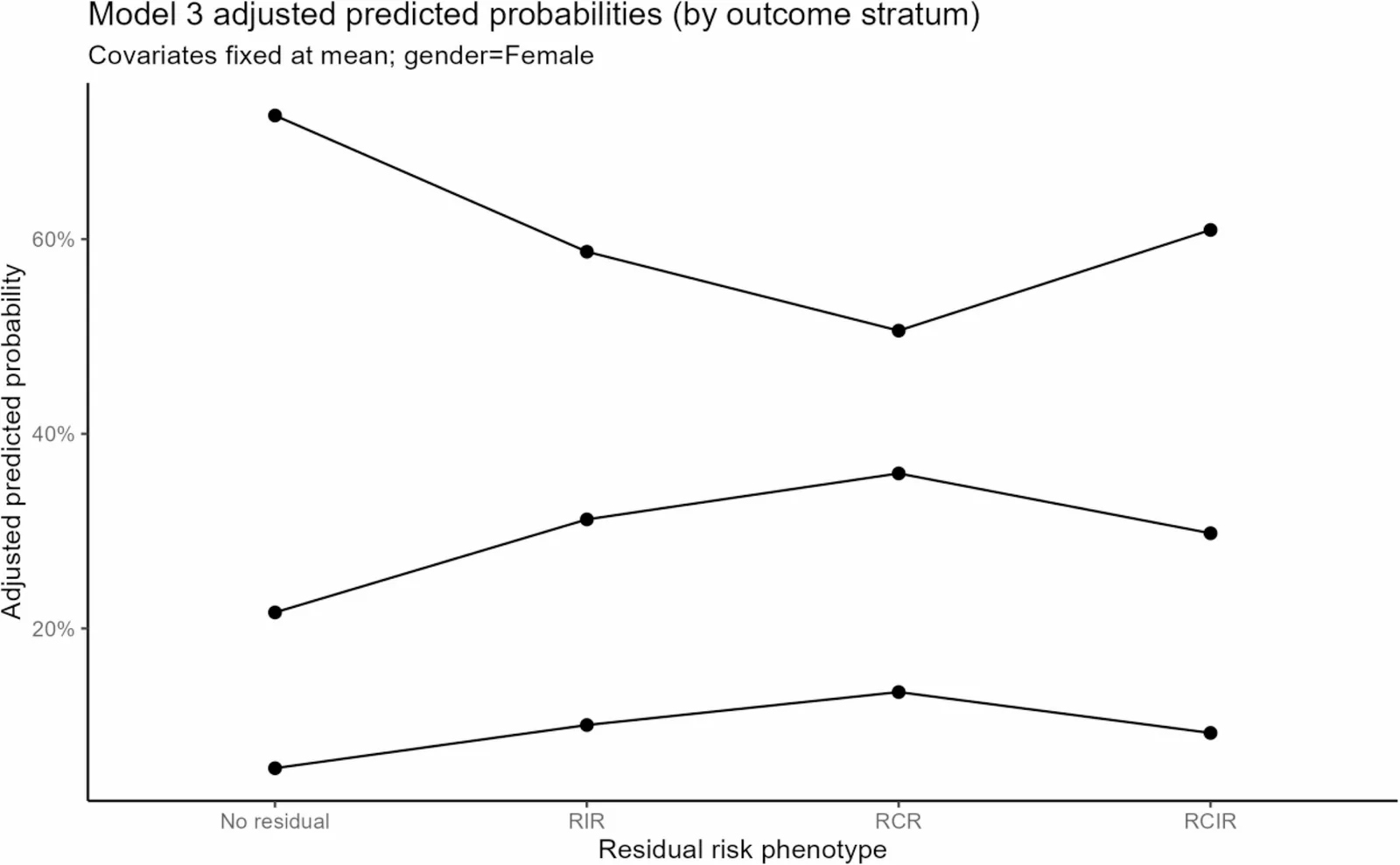
Fully adjusted (Model 3) marginal predicted probabilities of HFA-PEFF strata by residual risk phenotype. Model 3–adjusted marginal predicted probabilities of low (≤1), intermediate (2–4), and high (≥5) HFA-PEFF probability strata plotted by residual risk phenotype (no residual, RIR, RCR, RCIR). Model 3 adjusted for age, gender, BMI, SBP, GLU, and Scr. Predicted probabilities were computed with covariates fixed at their sample means; for visualization, gender was set to female (as labeled). The figure highlights persistence of phenotype ordering and probability redistribution under full cardiometabolic adjustment

Model 3-adjusted marginal predicted probabilities by phenotype are shown in Fig. 5  and Figure S2-S3. The overall ordering pattern across phenotypes remained largely preserved after additional cardiometabolic adjustment, indicating that the association between residual risk phenotypes and HFpEF probability categories was not fully explained by conventional cardiometabolic factors. 

Exploratory mediation analyses further assessed whether cardiometabolic components mediated the association between RCIR and high HFpEF probability (HFA-PEFF ≥ 5), using RCR as the reference (Table S4). No meaningful mediation was observed for SBP or BMI. Fasting glucose showed only a modest borderline mediation signal, whereas the association between RCIR and high HFpEF probability was not materially explained by the cardiometabolic mediators evaluated in this study.

## Discussion

In this cross-sectional analysis, inflammatory-lipid residual risk phenotypes were differentially associated with HFpEF probability categories, with combined residual inflammatory and cholesterol risk (RCIR) showing the closest alignment with higher HFpEF probability. These findings suggest that the coexistence of inflammatory activation and lipid-related residual risk may identify a clinically relevant phenotype beyond conventional cardiometabolic profiling.

HFpEF is increasingly regarded as a systemic syndrome characterized by the interplay of chronic low-grade inflammation, metabolic disturbance, endothelial dysfunction, and microvascular remodeling [22]. Within this framework, the joint presence of residual inflammatory activity and residual risk may reflect a biological milieu more compatible with myocardial stiffening, impaired relaxation, and progressive HFpEF-related remodeling. Persistent inflammation may potentiate lipid-related vascular injury through endothelial activation [23, 24], leukocyte recruitment [25, 26] and pro-fibrotic signaling [27], thereby contributing to microvascular rarefaction [28] and extracellular matrix remodeling [26], both of which are closely linked to HFpEF pathophysiology. Our findings extend this conceptual framework by suggesting that RCIR may serve as a marker of a higher-risk inflammatory-lipid phenotype associated with greater HFpEF likelihood, although the cross-sectional design precludes conclusions regarding temporality or causality.

An additional and potentially counterintuitive finding was that isolated RCR was more common in lower and intermediate probability categories, while remaining strongly associated with higher HFpEF probability in multivariable models [29]. Several explanations may account for this pattern. First, treatment-related confounding and reverse causation are plausible, as individuals with higher HFpEF probability may have been more likely to receive lipid-lowering therapy, thereby lowering LDL-C despite persisting cardiovascular risk [30-32]. Second, RCR may characterize an earlier cardiometabolic stage in which dyslipidemia is prominent, whereas inflammatory activation, structural remodeling, and overt HFpEF abnormalities are not yet dominant [33]. Third, because the phenotype assignment based on LDL-C and hs-CRP thresholds, group classification may also have been influenced by medication exposure, acute clinical context, and healthcare factors. Accordingly, the higher prevalence of RCR in lower probability categories should not be interpreted as protective, but rather as reflecting the dynamics and context-dependent nature of phenotype classification [13].

Adjustment for conventional cardiometabolic factors attenuated the observed associations to some extent, but did not fully account for them. In addition, exploratory mediation analyses did not support meaningful mediation through BMI or SBP, while fasting glucose showed only a modest borderline signal [34]. These findings suggest that the relationship between RCIR and higher HFpEF probability is not adequately captured by conventional measures of adiposity, blood pressure, or glycemic status alone. Rather than indicating a single direct biological pathway, the residual association may reflect broader processes not well represented by these routine indices, including systemic immune activation [22], endothelial dysfunction [24], microvascular inflammation [1], and tissue remodeling [35]. Given that all variables were measured contemporaneously, these mediation findings should be interpreted as exploratory and hypothesis-generating rather than mechanistic evidence.

### Clinical implications

These findings may have implications for HFpEF risk characterization. First, assessment based on lipid or metabolic indices alone may not fully capture higher HFpEF probability in individuals with persistent inflammatory activity. Second, integrated inflammatory-lipid residual risk phenotyping may be particularly informative in patients with intermediate HFA-PEFF probability, a group characterized by substantial heterogeneity and diagnostic uncertainty. In this setting, such phenotyping may help refine risk profiling and identify individuals who may benefit from closer clinical evaluation. Finally, our results provide a rationale for future studies to determine whether inflammatory-lipid residual risk phenotyping can improve HFpEF-oriented risk stratification beyond conventional cardiometabolic assessment.

### Limitations

Several limitations should be acknowledged. First, the cross-sectional design precludes determination of temporal sequence and limits causal interpretation. Although multivariable adjustment and sensitivity analyses were performed, residual confounding cannot be excluded, particularly with respect to medication exposure, treatment indication, and acute clinical status. Second, the HFA-PEFF score was used to classify HFpEF probability rather than to establish a definitive diagnosis, and some degree of misclassification across probability categories is possible. Third, the phenotype definitions were based on LDL-C and hs-CRP thresholds measured during hospitalization and may have been influenced by treatment, concurrent clinical conditions, and other unmeasured contextual factors. Fourth, the mediation analyses were exploratory and restricted to selected conventional cardiometabolic mediators, whereas other potentially relevant intermediates were not available. Accordingly, the present findings should be interpreted as hypothesis-generating and confirmation in prospective studies.

### Conclusion

In conclusion, inflammatory-lipid residual risk phenotypes are differentially associated with HFA-PEFF probability categories. RCIR was more closely aligned with higher probability, and this association was not fully explained by the conventional cardiometabolic factors examined in this study. These findings support the potential value of inflammatory-lipid residual risk phenotyping in refining HFpEF risk characterization and provide a basis for future prospective studies.

## Supplementary Information


Supplementary Material 1.


## Data Availability

The datasets generated and analyzed during the current study are not publicly available due to privacy restrictions but are available from the corresponding author on reasonable request.

## Abbreviations

BMI: body mass index
Scr: serum creatinine
GLU: fasting glucose
hs-CRP: high-sensitivity C-reactive protein
LDL-C: low-density lipoprotein cholesterol
SBP: systolic blood pressure
