# Supplementary material for: Inflammatory–lipid residual risk phenotypes delineate the HFpEF probability spectrum independent of cardiometabolic mediation
Source: BMC Cardiovasc Disord. 2026 Apr 29;26:757. doi: 10.1186/s12872-026-05836-3 (PMC13528071; doi:10.1186/s12872-026-05836-3)
Supplement: Supplementary file 1 [file 12872_2026_5836_MOESM1_ESM.docx]

**Table S1-4.**

**Table S1. Sensitivity analyses excluding participants with hs-CRP >10 mg/L.**

| model | comparison | OR | CI_low | CI_high | P_value |
| --- | --- | --- | --- | --- | --- |
| Model 3 | RIR vs No residual | 0.44 | 0.18 | 1.07 | 0.069 |
| Model 3 | RCR vs No residual | 0.38 | 0.23 | 0.67 | <0.001 |
| Model 3 | RCIR vs No residual | 0.55 | 0.31 | 0.97 | 0.039 |

Sensitivity analysis of the fully adjusted ordinal logistic regression model after excluding participants with markedly elevated hs-CRP levels (>10 mg/L). Results are presented as odds ratios (ORs) with 95% confidence intervals (CIs) for residual inflammatory risk (RIR), residual cholesterol risk (RCR), and combined residual cholesterol and inflammatory risk (RCIR), using the no-residual-risk phenotype as the reference group.

**Table S2. Binary logistic regression using alternative HFpEF probability thresholds.**

| **model** | **comparison** | **OR** | **CI_low** | **CI_high** | **P_value** |
| --- | --- | --- | --- | --- | --- |
| Model1 | RIR vs No residual | 0.86 | 0.31 | 2.32 | 0.76 |
| Model1 | RCR vs No residual | 0.33 | 0.14 | 0.67 | 0.004 |
| Model1 | RCIR vs No residual | 0.80 | 0.34 | 1.64 | 0.57 |
| Model2 | RIR vs No residual | 0.28 | 0.08 | 0.92 | 0.04 |
| Model2 | RCR vs No residual | 0.36 | 0.13 | 0.85 | 0.03 |
| Model2 | RCIR vs No residual | 0.71 | 0.26 | 1.73 | 0.47 |
| Model3 | RIR vs No residual | 0.29 | 0.08 | 0.99 | 0.05 |
| Model3 | RCR vs No residual | 0.31 | 0.11 | 0.75 | 0.02 |
| Model3 | RCIR vs No residual | 0.60 | 0.21 | 1.49 | 0.30 |

Binary logistic regression models using alternative dichotomizations of HFA-PEFF probability. Model covariates are consistent with the main analysis where applicable. Reported as ORs with 95% CIs.

**Table S3. Multinomial logistic regression models for HFA-PEFF probability categories.**

| **Group** | **term** | **estimate** | **std.error** | **P** | **CI_low** | **CI_high** |
| --- | --- | --- | --- | --- | --- | --- |
| Intermediate | Intercept | 0.00 | 0.01 | 0 | 0.00 | 0.00 |
| Intermediate | RIR | 0.65 | 0.08 | 0.00 | 0.56 | 0.75 |
| Intermediate | RCR | 1.03 | 0.10 | 0.73 | 0.85 | 1.25 |
| Intermediate | RCIR | 1.45 | 0.09 | 0.00 | 1.20 | 1.74 |
| Intermediate | Age | 1.07 | 0.01 | 0.00 | 1.06 | 1.09 |
| Intermediate | Gender_Male | 0.97 | 0.15 | 0.82 | 0.73 | 1.29 |
| Intermediate | BMI | 1.01 | 0.01 | 0.57 | 0.98 | 1.04 |
| Intermediate | SBP | 1.01 | 0.00 | 0.00 | 1.01 | 1.02 |
| Intermediate | GLU | 1.05 | 0.02 | 0.03 | 1.01 | 1.10 |
| Intermediate | Cr | 0.10 | 0.00 | 0.53 | 1.00 | 1.00 |
| High | Intercept | 0.00 | 0.01 | - | 0.00 | 0.00 |
| High | RIR | 0.37 | 0.08 | 0.00 | 0.31 | 0.44 |
| High | RCR | 0.35 | 0.10 | 0.00 | 0.29 | 0.43 |
| High | RCIR | 0.69 | 0.10 | 0.00 | 0.57 | 0.83 |
| High | Age | 1.13 | 0.01 | 0.00 | 1.12 | 1.15 |
| High | Gender_Male | 0.93 | 0.15 | 0.60 | 0.70 | 1.23 |
| High | BMI | 1.01 | 0.01 | 0.49 | 0.98 | 1.04 |
| High | SBP | 1.02 | 0.00 | 0.00 | 1.01 | 1.02 |
| High | GLU | 1.09 | 0.02 | 0.00 | 1.04 | 1.14 |
| High | Cr | 1.00 | - | 0.95 | 1.00 | 1.00 |

Multinomial logistic regression using HFA-PEFF probability strata as a three-level outcome to relax the proportional-odds assumption. Model 3 covariates include age, gender, BMI, SBP, GLU, and Cr. Results are reported as relative ORs with 95% CIs.

**Table S4. Exploratory mediation analyses of cardiometabolic components.**

| Mediator | Effect | Estimate | 95% CI | P value |
| --- | --- | --- | --- | --- |
| SBP | ACME | 0.0004 | -0.0021–0.0034 | 0.73 |
|  | Proportion Mediated | 0.4% | -3.0%–4.6% | 0.73 |
|  | ADE | 0.0829 | 0.0408–0.1264 | <0.001 |
|  | Total Effect | 0.0833 | 0.0404–0.1265 | <0.001 |
| BMI | ACME | -0.0001 | 0.0012–0.0011 | 0.964 |
|  | Proportion Mediated | -0.06% | -1.76%–1.37% | 0.964 |
|  | ADE | 0.0828 | 0.0406–0.1266 | <0.0001 |
|  | Total effect | 0.0828 | 0.0404–0.1265 | <0.0001 |
| GLU | ACME | 0.0027 | -0.0000–0.0062 | 0.056 |
|  | Proportion Mediated | 3.1% | -0.0%–8.7% | 0.056 |
|  | ADE | 0.0829 | 0.0406–0.1264 | <0.0001 |
|  | Total effect | 0.0855 | 0.0428–0.1296 | <0.0001 |

Exploratory mediation analyses evaluating whether systolic blood pressure, body mass index, or fasting glucose mediated the association between RCIR and high HFpEF probability (HFA-PEFF ≥5), using RCR as the reference group. Average causal mediation effects (ACME), average direct effects (ADE), total effects, and proportions mediated are reported with 95% bootstrap confidence intervals based on 1,000 simulations. These mediation analyses were considered hypothesis-generating.

**Figure S1-3.**

**Figure S1. Flow diagram of participant selection.**

Flow diagram showing the screening, eligibility assessment, exclusions, and final inclusion of participants in the analytical cohort. Participants were required to have sufficient clinical, laboratory, and echocardiographic data for HFA-PEFF scoring and inflammatory-lipid residual risk phenotyping.

**Figure S2. Predicted probabilities of HFA-PEFF categories across residual risk phenotypes under Models 1–3.**

Stacked bar plots showing the marginal predicted probabilities of low, intermediate, and high HFA-PEFF categories across residual risk phenotypes under Model 1 (unadjusted), Model 2 (adjusted for age and gender), and Model 3 (further adjusted for BMI, systolic blood pressure, fasting glucose, and serum creatinine). The figure illustrates the overall redistribution of HFA-PEFF probability across phenotypes after sequential adjustment. Covariates were fixed at sample means for visualization, and gender was set to female.

**Figure S3. Model 3-adjusted predicted probabilities of HFA-PEFF categories by residual risk phenotype.**

 Stacked bar plots showing the marginal predicted probabilities of low, intermediate, and high HFA-PEFF categories across residual risk phenotypes derived from the fully adjusted ordinal logistic regression model (Model 3). Model 3 was adjusted for age, gender, BMI, systolic blood pressure, fasting glucose, and serum creatinine.
